# Revealing Distress and Perceived Stress among Dentists at the Outset of the COVID-19 Pandemic: A Cross-Sectional Factor Analytic Study

**DOI:** 10.3390/ijerph182211813

**Published:** 2021-11-11

**Authors:** Kenneth S. Serota, Bálint Andó, Katalin Nagy, Ildikó Kovács

**Affiliations:** 1 Doctoral School of Clinical Medicine, Albert Szent-Györgyi Medical School, University of Szeged, H-6720 Szeged, Hungary; kendo4310@gmail.com; 2 Department of Psychiatry, Albert Szent-Györgyi Medical School, University of Szeged, H-6720 Szeged, Hungary; ando.balint@med.u-szeged.hu; 3 Department of Oral Surgery, Faculty of Dentistry, University of Szeged Tisza L. Krt, H-6720 Szeged, Hungary

**Keywords:** perceived stress, psychological distress, COVID-19, dentists

## Abstract

Dentists’ perceptions about the stressfulness of clinical practice are well-documented, but literature on perceived stress and psychological distress experienced during the COVID-19 pandemic is scarce. This study aims to explore the emotions and attitudes, and the socio-demographic, dental, and COVID-related factors that are associated with the emergence of perceived stress and psychological distress that have been experienced by dentists during the COVID-19 pandemic. General demographic and dental-related data, and specific questions measuring the potential factors regarding dental professionals’ concerns and opinions about their professional circumstances during the pandemic, were electronically collected from 182 dental practitioners. Exploratory and confirmatory factor analyses were used to assess whether dentists’ emotions and attitudes during the pandemic measure the same construct: psychological distress, while linear regression models were built on the exploration of the effects of COVID-related factors on perceived stress and psychological distress. Facets of impulsiveness, lack of interest in social connections, emotional disengagement, mood swings, and acknowledgment of emotional exhaustion due to the pandemic, were measurements of the same construct and manifested in a singular factor: psychological distress. Two aspects, the fear of aerosol propagation and insecurities of financial status, increased the likelihood of the emergence of heightened levels of perceived stress and distress, while years spent in dental practice and age seemed to be protective factors against perceived stress and distress.

## 1. Introduction 

At the end of 2019, in the Wuhan province of China, the spread of a new virus of the family Coronaviridae was detected. By March 2020, the rapid, global spread of SARS-CoV-2 (COVID-19) caused the World Health Organization (WHO) to categorize the pandemic as a public health emergency [1,2] during which, in contrast with public health guidance for previous pandemics [3,4], governments mandated dentists to postpone elective dental procedures and to concentrate solely on emergency patient care. 

In general, dentists are known to function under stressful conditions [5,6], hence the need to identify risk factors that could impact upon the levels of psychological symptoms, such as the levels of perceived stress and distress experienced by dentists due to the unprecedented challenge of the COVID-19 pandemic, is in high demand. Dentists have already been shown to have elevated levels of subjective overload and psychological distress, which differed among countries due to social, cultural, and environment issues [7]. However, despite these differences, the assessment of psychological aspects, such as stress, in dental professionals is of high interest. The spread of the COVID-19 pandemic recently highlighted those major concerns and stressors that influence the different aspects of the professional and personal lives of dentists [8,9]. Complications for the dentistry profession may also have further, unknown, long-term impacts on clinical practice, dental education, and dental research [10,11]. 

Perceived stress represents the feelings or thoughts that an individual has about the level of stress they are under at a given point in time or over a given time period [12], while psychological distress can be regarded as a mental health condition characterized by a complex set of emotional/psychological symptoms, i.e., various somatic and psychological symptoms, such as the elevated levels of anxiety, depression or the feeling of burnout, and is accompanied with several stress-related, socio-demographic, and work-related risk factors [13].

Dentists are habitually at high risk of contagion and transmission of infection because of direct and indirect contact with blood, saliva and other biologic fluids in the oral cavity and airways [14], as well as the presence of bacteria and viruses in the aerosols created by dental procedures [15]. The instruments used in dental procedures produce significant aerosol dispersion [16,17]; therefore, direct and indirect contact transmission from particulate matter (PM), droplet inhalation, and fluid transfer would put dentists, team members and, indirectly, their families, at high risk of contagion [18,19].

Alterations to the dental facility are essential to remove PM dispersion. High levels of PM are shown to occur with the use of natural ventilation, as dental treatment rooms are typically of an open design. High-volume evacuation filters (HVE) and high-efficiency particulate-absorbing filtration (HEPA) are effective at removing PM; however, HVE requires the presence of auxiliary personnel and HEPA filters require constant maintenance to ensure that the unit does not become a source of pathogens [20,21]. The professional must continually strive to minimize the impact of aerosol-generating procedures and mitigate the possibility of bidirectional disease transmission [22].

As an occupational hazard, the risk of exposure to sources of infection, such as the human immune deficiency virus (HIV), tuberculosis (TB), and the hepatitis B virus (HBV), invariably heighten anxiety and work stressors [23]. Elevated levels of perceived risk are a function of the uncertainty of infection regardless of patient triage as the SARS-CoV-2 virus can spread from asymptomatic, symptomatic, and pre-symptomatic patients [24]. The use of a rubber dam, social distancing in treatment areas, and structural changes to a facility were essential in reducing contagion among dentists and patients. The use of teledentistry and online triage prior to appointments were shown to be of significant value in managing risk factors of infection. Despite these modifications and the use of improved personal protective equipment (PPE), dentists remained fearful of infection during treatment procedures [25].

Psychological variables, such as poor social support and a lack of self-efficacy, were reported as significant risk and contributory factors for the emergence of perceived stress and psychological distress in healthcare workers [26]. Based on the economic consequences of past pandemics, a global recessionary trend is to be expected as a result of travel restrictions, social distancing, and quarantine [27]. The career-related uncertainty posed by the novel virus could heighten levels of perceived stress and psychological distress [28,29] among dentists, who are in a profession identified as one of the most significant for stress, burnout, anxiety, and depression [30].

The literature on dentists and perceived stress primarily addresses those in training [31,32,33]. Furthermore, the literature on perceived stress and distress connected to the pandemic is scarce among licensed dental professionals [34]. The aim of the present study was to comprehensively assess the phenomenon of stress among dental professionals: to identify the possible risk factors behind the emergence of perceived stress and psychological distress, and to assess the contribution of these factors to them in dentists during the COVID-19 pandemic.

## 2. Materials and Methods

### 2.1. Participants and Procedure

Licensed dentists who are members of the Hungarian Dental Association were invited to answer an electronic test battery, to gather information about dentists’ attitudes, emotions, and information about their opinions regarding their practice after severe acute respiratory syndrome-Coronavirus-2 (SARS-CoV-2) lockdown. A total of 208 dentist replied. A total of 26 participants were excluded from final analysis; thus, 182 participants’ data were evaluated. The study was conducted in accordance with the Declaration of Helsinki, and it was approved by the Human Investigation Review Board, University of Szeged (ethical approval number: 27-XXVII-061-1/2021). Informed consent from each participant was also acquired online prior to participation in the survey.

### 2.2. Measures

The first section of the survey is composed of questions regarding demographic information: age, gender, residence, years of being a dental practitioner, place of practice, marital status, number of children, and financial status. In the second section, dentists filled out the Perceived Stress Scale, which is a self-assessment questionnaire including 14 questions to be rated on a 5-point Likert scale that reflected on stressful experiences in the past months [35]. The Hungarian adaptation proved to be valid and reliable [36]; internal consistency of the PSS on our sample was excellent (Cronbach’s α = 0.926).

Additionally, four specific questions were formed to measure potential factors regarding dental professionals’ concerns and opinions about the likelihood of infection and their professional futures in relation to COVID-19 and its potential consequences (see Table 1). Questions could be rated on a 5-point Likert scale ranging from 1—I disagree completely, 2—I disagree, 3—I neither agree nor disagree, 4—I agree to 5—I agree completely. Moreover, 12 questions regarding dentists’ emotions and attitude towards the SARS-CoV-2 pandemic were included. Items were rated on a 5-point Likert scale ranging from 1—never, 2—rarely, 3—sometimes, 4—often to 5—always (for the complete list of questions, see Table 2).

### 2.3. Data Analysis

To explore whether the questions posed to dentists, regarding their emotions and attitude during the pandemic, measure the same construct (psychological distress). An exploratory factor analysis (EFA) with principal component analysis was used to test the structure of the presupposed Distress Factor; varimax rotation was selected to determine the factors under which each item loads. To identify the number of factors to be retained in the final model, the Kaiser method of factor retention, the evaluation of eigenvalues above 1, and a screen test were utilized. Item factor loadings were also evaluated, where a cutoff of 0.40 was applied for item inclusion. The goodness of fit of the components of the Distress Factor was tested with confirmatory factor analysis. The models were estimated through covariance matrices with the use of maximum likelihood estimation. Model fitness was evaluated by the following fit indices: chi-square (χ^2^), absolute, and incremental fit indices (RMSEA, TFI and CFI, respectively) and a parsimonious fit index (AIC). Since the χ^2^ value is sensitive to sample size, i.e., in case of larger samples, it is likely that reasonable models also result in statistically significant χ^2^ and *p* values [37,38,39,40], and thus previously mentioned fit indices were also considered (Byrne, 2005). The determination of a good fit is based on the following recommendation: CFI is above 0.95 and for RMSEA, above 0.06 [41]. The result of the factor analyses was used to create a new variable that measures dentists’ distress during the COVID-19 pandemic. To examine the effects of demographic variables, dental-specific questions on the likelihood that dentists experience perceived stress, and the hypothesized distress factor, linear logistic regressions were used with the stepwise regression method. Analyses were conducted with IBM SPSS v24 [42] and IBM AMOS v23 [43].

Dentists experienced psychological symptoms connected to the pandemic, which were predominantly restlessness, annoyance, sleep disturbance, and mood swings. The complete list of survey questions and descriptive statistical data are listed in Table 2. 

## 3. Results

A total of 182 licensed dentists completed the survey and their data were further analyzed. Participants were between 24 and 81 years (mean: 50.93; SD: 13.67) with an average of almost 26 years spent working in a dental practice. Table 1 indicates detailed descriptive demographic characteristics of the sample.

The first 12 items were explored to see whether they measure the same a priori construct: psychological distress connected to the pandemic. Thus, to test whether these measured constructs fit the hypothesized measurement model, both an exploratory factor analysis (EFA) and a confirmatory factor analysis (CFA) were used.

### 3.1. Exploratory Factor Analysis

The sample size of the present study was sufficient for the analyses, since 182 participants filled out the 12-item survey, which fulfils the recommended 10:1, participant:item ratio [39]. Evaluation of the correlation matrix was significant in each case between item correlations of 0.113 to 0.710, Kaiser–Meyer–Olkin Measure of Sampling Adequacy (KMO = 0.909) and, the Bartlett’s test of sphericity (χ^2^(66) = 834.092, *p* ≤ 0.001) suggested that these items are connected but show enough variability to proceed with the principal component analysis. The Kaiser method of factor retention identified two factors with eigenvalues above 1, and a scree test for factor retention suggested a one-factor model; thus, the final model included one factor [44] (see Table 3). 

The final model included one factor with all 12 items loading on one factor, with at least 0.441 loadings; communalities for each item were all moderate to high (see Table 4).

### 3.2. Confirmatory Factor Analysis

**Figure 1:**
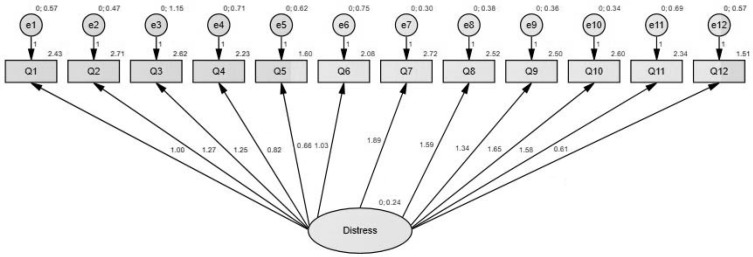
Confirmatory factor analysis representing the latent variable (distress factor). The ellipse represents the latent variable (distress factor), boxes depict observed variables as follows: Q1: I am preoccupied by things that are not at all or slightly worrying; Q2: I get annoyed easily; Q3: I have trouble sleeping; Q4: I am indifferent towards various activities; Q5: I have barely any or no appetite; Q6: I feel emotionally distant from others; Q7: I feel restless; Q8: I feel sad; Q9: I feel angry; Q10: I have mood swings; Q11: I am noticing the signs of burnout; Q12: I employ addictive behaviour (smoking, drugs, alcohol) to cope with stress. Arrows pointing towards each observed variable represent factor loadings, while the values above each observed variable depict the explained variance.

The model was re-estimated with confirmatory factor analysis to assess model fitness. The fit indices of the one-factor model proved to be acceptable: *Χ*^2^ = 92.447, df = 54, *Χ*^2^/df = 1.712, *p* ≤ 0.001, CFI = 0.952, TLI = 0.931, RMSEA = 0.063, AIC = 164.447 (see Figure 1).

Based on this, we created a new variable representing the standardized values of the psychological distress factor, where 0 refers to the convergence to the sample mean, negative values stand for lower level of distress, and positive values represent a higher level of experienced distress. Internal consistency on our sample was great (Cronbach’s α = 0.876).

### 3.3. Effects of COVID-Related Variables on Perceived Stress and Psychological Distress

Two stepwise linear regression models were built on exploring the effects of COVID-related factors on perceived stress and the result of the factor analyses: psychological distress. The first linear regression was carried out to evaluate whether the following predicted perceived stress: age, gender, residence, place of practice, marital status, number of children, financial status compared to national average, whether they felt that the COVID-19 pandemic will have long-lasting consequences on the financial situation of their practice, whether they felt that phone or online consultations would remain an integral part of their practice even after the COVID-19 pandemic would be under control, whether they felt that not even the strict compliance with professional rules can fully resolve the issues caused by aerosol propagation, and whether they felt that their personal professional development is facilitated by online education until the pandemic is significantly under control. The results of the regression model indicated that the final model explained 22.9% of the variance and was significant (F (1162) = 15.149, *p* ≤ 0.001). It was found that the following significantly predicted the level of perceived stress: financial status, number of years in practice, and the feeling that not even strict compliance with professional rules can fully resolve the issues caused by aerosol propagation.

The second linear regression was carried out to evaluate whether the following significantly predicted perceived psychological distress: age, gender, residence, place of practice, marital status, number of children, financial status compared to national average, whether they felt that the COVID-19 pandemic will have long-lasting consequences on the financial situation of their practice, whether they felt that phone or online consultations would remain an integral part of their practice even after the COVID-19 pandemic would be under control, whether they felt that not even the strict compliance with professional rules can fully resolve the issues caused by aerosol propagation, and whether they felt that their personal professional development would be facilitated by online education until the pandemic is under control. The results of the regression model indicated that the final model explained 14.9% of the variance and was significant (F (1163) = 7.496, *p* = 0.007. It was found that the following significantly predicted the level of psychological distress: age, financial status, and the feeling that not even the strict compliance with professional rules can fully resolve the issues caused by aerosol propagation (see Table 5).

## 4. Discussion

The aim of this study was to reveal the background and potential risk factors of the level of perceived stress and psychological distress associated with the impact of COVID-19 among dentists. This work investigated the role of socio-demographic variables (gender, age, residence, years in practice, place of dental practice, marital status, number of children, and financial status) to ascertain which factors contribute to the appearance of elevated levels of perceived stress and psychological distress in connection with the COVID-19 lockdown.

Factor analysis was used to ascertain whether questions regarding dentists’ emotions and attitude during the pandemic measured the same construct: psychological distress. The present work points out that the psychological distress manifested in one factor, consisting of features of impulsiveness, lack of interest in social connections, emotional disengagement, mood swings, and acknowledgment of emotional exhaustion because of the pandemic. There are ample studies showing that dentists are prone to significant psychological distress due to the intense mental health stressors of clinical practice [45,46]. There is a paucity of the current literature addressing perceived stress (the lack of control and unpredictability in response to stressors) because of the pandemic [47,48].

The results indicated that aerosol propagation during dental procedures significantly promoted psychological symptoms, such as the elevated level of perceived stress and distress among dentists. In line with this, an in vitro study showed that the SARS-CoV-2 virus remained viable in aerosols for a minimum of 3 h with median estimates of half-life between 1.1 to 1.2 h [49]. Moreover, the virus persistently adheres and remains infectious from 2 h up to 9 days on various surfaces [50]. New infection control protocols are needed to mitigate the possibility of cross-contamination during and after the COVID-19 pandemic. 

At the time of the present study, there were a lack of data about the SARS-CoV-2 virus transmission vectors in the dental office. Infection control measures were in a state of flux [51]: shortfalls in personal protective equipment (PPE) resulted in an anxiety regarding the heightened perceived stress of risk and consequence. This was further exacerbated by guidelines from government agencies regarding the nature and the mitigation of the virus [52]. 

It has been reported in the literature that acute COVID-19 infection could contribute to adverse outcomes concerning oral health due to impairment of the immune system. As shown by Dziedic and Wojtyczka, this could lead to xerostomia, opportunistic fungal infections, dysgeusia, unspecific oral ulcerations, recurrent oral herpes simplex (HSV-1), and ulcerative gingivitis due to the increase in susceptibility of the oral mucosa [53]. To date, while there is no scientific evidence that certifies which oral symptoms SARS-CoV-2 can cause, it is reasonable to assume that the oral manifestations described could be a warning sign of the presence of viral load [54]. 

The study found that uncertainty regarding income disruption was a significant contributing factor to perceived stress and psychological distress. Financial stress and anxiety are contributory factors to cognitive dysfunction, which could amplify levels of perceived stress [55]. While dentists did feel a moral and professional obligation to care for their patients, the financial consequences of practice closure in the short- and long-term were seen as an a priori source of psychological distress [56]. 

In countries throughout the world, diminished production and lost income due to government-mandated stoppage of elective treatment caused high levels of distress, due to the costs to restructure a practice physical facility, the need to replace PPE after each patient, and stringent infection controls that reduced the number of available appointment times [57]. The pandemic has irrevocably altered the practice of dentistry. Opportunities are available for dental researchers to focus on the cost/benefit ratio of expanded PPE use and other changes in workflows that could be of significance in the mitigation of perceived stress and distress in a workplace environment in the future. Those in solo private practice [58] experienced higher levels of distress than those dentists in governmental hospitals or institutions [59,60] as their earning potential, while greater, is not contractual [61].

Regarding age and years spent in clinical practice, results of this work conformed to the previous literature [62], since these factors have been shown to be protective against perceived stress and psychological distress. Regarding time in practice as a function of perceived stress and distress, the biologic and economic problems associated with the current pandemic were not evident in previous pandemics, particularly among healthcare providers [63,64].

Dental professionals with more work experience were less likely to report fear of changes in the work environment, fear of being infected and infecting others [65]. Less work-related distress could be explained by increased self-confidence among dentists with more work experience [66]. Given the fluctuating dynamics of a dental career, younger clinicians are particularly susceptible to perceived stress [67] and psychological distress, due to concerns regarding fewer job prospects. As repeated lockdowns have been necessary through 2020 and 2021 due to surges in case numbers and variants of the virus, the continued effect on the global economy could sustain increasing levels of anxiety, perceived stress, and distress amongst dentists of any age and gender as to their future vision of their careers [68].

This study has several limitations. The data were self-reported during the early months of the pandemic. As the dynamic nature of the pandemic was associated with repeated modifications of infection controls, it is important to consider the knowledge level of participants in this study within the time frame of response to the questionnaire. Another limitation is that the present study did not incorporate gender disaggregation, for which further studies with increased sample sizes would be necessary to underscore the robustness of the identified distress factor by gender disaggregated analysis. Additionally, further research will be necessary to determine the perceived stress and distress associated with the long-term impact of the financial, psychological, and professional outcomes of the pandemic, especially as the pandemic continues unabated. It is reasonable to assume that this could be of concern to dentists both currently and after the control of the spread and contagion of the pandemic. [69,70].

## 5. Conclusions

This study provided an understanding of two aspects of stress, perceived stress and psychological distress, among dentists during the COVID-19 pandemic. Psychological distress was operationalized by features reflecting on impulsiveness, lack of interest in social connection, emotional disengagement, mood swings, and acknowledgment of emotional exhaustion. Two major risk factors were identified in both aspects of stress: the fear of aerosol propagation and concerns about financial status. Fear of aerosol propagation and insecurities of financial status both increased the likelihood of the emergence of heightened levels of perceived stress and distress. Years spent in dental practice and age seemed to be protective factors against perceived stress and distress. Since dental professionals are proven to be highly prone to distress and perceived stress, measures should be taken that ascertain the factors that are likely to elevate the level of these psychological symptoms.

The increasing frequency of global pandemics since the turn of the century warrants aggressive iterations of PPE equipment and modified treatment protocols to minimize aerosol propagation. It is advisable for the dentistry profession to provide mental health support systems for clinicians to mitigate the effects of perceived stress and psychological distress in the long term.

## Figures and Tables

**Table 1:** Demographic characteristics of the study sample of dental practitioners.

Characteristic	N	%
**Gender**		
Male	62	34.1
Female	120	65.9
**Age (SD)**	50.95 (13.67) (min–max: 24–81 years)	
**Residence**		
Capital city	48	26.4
City	117	64.3
Town	5	2.7
Village	11	6.0
**Years of being a dental practitioner (SD)**	25.90 (13.63) (min–max: 0–57 years)	
**Place of dental practice**		
Capital city	50	27.5
City	106	58.9
Town	12	6.7
Village	12	6.7
**Marital status**		
Married/In domestic partnership	145	79.7
Single	33	18.1
**Number of Children**		
0	36	19.8
1	39	21.4
2	74	40.7
3	25	13.7
4	4	2.2
5	2	1.1
**Financial status compared to national average**		
significantly better	20	11.0
better	111	61.0
similar	43	23.6
worse	6	3.3
significantly worse	1	0.5

**Table 2:** Descriptive statistics of survey questions.

Question Regarding Emotions and Attitudes towards COVID-19	Mean (SD)
I am preoccupied by things that are not at all or slightly worrying	2.44 (0.902)
I get annoyed easily	2.71 (0.903)
I have trouble sleeping	2.62 (1.241)
I am indifferent towards various activities	2.23 (0.936)
I have barely any or no appetite	1.60 (0.853)
I feel emotionally distant from others	2.08 (1.008)
I feel restless	2.73 (1.082)
I feel sad	2.52 (1.001)
I feel angry	2.50 (0.897)
I have mood swings	2.60 (1.002)
I am noticing the signs of burnout	2.34 (1.144)
I employ addictive behaviour (smoking, drugs, alcohol) to cope with stress	1.51 (0.814)
**Question regarding dental professionals’ concerns and opinions**	**Mean (SD)**
I feel that the COVID-19 pandemic will have long-lasting consequences on the financial situation of my practice.	3.53 (1.016)
I feel that phone or online consultations will remain an integral part of my practice even after the COVID-19 pandemic will be under control.	3.21 (1.128)
I feel that not even the strict compliance with professional rules can fully resolve the issues caused by aerosol propagation.	3.99 (1.049)
I feel that my personal professional development is facilitated by online education until the pandemic gets under control.	3.35 (1.098)

**Table 3:** Eigenvalues, extraction loadings and variances of one-factor and two-factor models.

Factor	Initial Eigenvalues			Extraction Sums of Squared Loadings			Rotation Sums of Squared Loadings		
	Total	Variance%	Cumulative%	Total	Variance%	Cumulative%	Total	Variance%	Cumulative%
1	5.275	43.958	43.958	5.275	43.958	43.958	4.088	34.070	34.070
2	1.148	9.964	53.522	1.148	9.564	53.522	2.334	19.452	53.522

**Table 4:** Final factor loadings and communalities.

Items	Factor Loadings	Communalities
I feel restless	0.847	0.727
I have mood swings	0.811	0.705
I feel sad	0.798	0.638
I feel angry	0.758	0.665
I am noticing the signs of burnout	0.729	0.532
I get annoyed easily	0.689	0.672
I am preoccupied by things that are not at all or slightly worry	0.613	0.378
I feel emotionally distant from others	0.566	0.325
I have trouble sleeping	0.563	0.518
I am indifferent towards various activities	0.512	0.313
I have barely any or no appetite	0.460	0.642
I employ addictive behaviour (smoking, drugs, alcohol) to cope with stress	0.441	0.308

**Table 5:** Effects of COVID-related factors on perceived stress and psychological distress.

1st Regression	B	Std. Error	β	t	sig.	Tolerance	VIF
aerosol propagation	0.501	0.614	0.283	4.075	≤0.001	0.987	1.014
financial status	3.558	0.914	0.272	3.892	≤0.001	0.977	1.024
years in dental practice	−0.216	0.048	−0.314	−4.449	≤0.001	0.978	1.022
**2nd Regression**							
aerosol propagation	0.240	0.070	0.249	3.429	0.001	0.987	0.013
financial status	0.346	0.105	0.242	3.296	0.001	0.969	1.032
age	−0.015	0.005	−0.201	−2.738	0.007	0.972	1.029

## Data Availability

The data presented in this study are available on request from the corresponding author. The data are not publicly available due to privacy.
